# Morbid obesity impacts mortality among inpatients with type a aortic dissection: an analysis of the national inpatient sample

**DOI:** 10.1186/s13019-022-02080-6

**Published:** 2023-01-10

**Authors:** Xiao Xu, Renqi Yin, Kangkang Zhi, Yingyi Qin, Boxiang Tu, Shengyong Wu, Ziwei Dong, Dongxu Liu, Jia He

**Affiliations:** 1 Tongji University School of Medicine, Shanghai, 200092 China; 2 Department of Vascular Surgery, Changzheng Hospital, Naval Medical University, Shanghai, 200433 China; 3 Department of Military Health Statistics, Naval Medical University, Shanghai, 200433 China

**Keywords:** Type A aortic dissection, Morbid obesity, In-hospital mortality, Prevalence

## Abstract

**Background:**

Stanford type A aortic dissection (T(A)AD) is one of the most dangerous cardiovascular diseases and morbid obesity is associated with the prognosis of many cardiovascular diseases. The aim of this study is to investigate the impact of morbid obesity on in-hospital mortality, total hospital costs and discover the prevalence of morbid obesity among inpatients with T(A)AD.

**Methods:**

Patients with a primary diagnosis of T(A)AD were identified from the National Inpatient Sample database (NIS) from 2008 to 2017. These patients were categorized into non-obesity, obesity and morbid obesity. Multivariable regression models were utilized to assess the association between obesity/morbid obesity and in-hospital mortality, total cost and other clinical factors. The temporal trend in prevalence of obesity/morbid obesity in T(A)ADs and the trend of in-hospital mortality among different weight categories were also explored.

**Results:**

From the NIS database 8489 T(A)AD inpatients were identified, of which 7230 (85.2%) patients were non-obese, 822 (9.7%) were obese and 437 (5.1%) were morbid obese. Morbid obesity was associated with increased risk of in-hospital mortality (odds ratio [OR] 1.39; 95% confidence interval [CI] 1.03–1.86), 8% higher total cost compared with the non-obese patients. From 2008 to 2017, the rate of obesity and morbid obesity in patients with T(A)AD have significantly increased from 7.36 to 11.33% (*P* < 0.001) and from 1.95 to 7.37% (*P* < 0.001). Factors associated with morbid obesity in T(A)ADs included age, female, elective admission, hospital region, dyslipidemia, smoking, rheumatoid arthritis/collagen vascular diseases, chronic pulmonary disease, diabetes and hypertension.

**Conclusions:**

Morbid obesity are connected with worse clinical outcomes and more health resource utilization in T(A)AD patients. Appropriate medical resource orientation and weight management education for T(A)AD patients may be necessary.

**Supplementary Information:**

The online version contains supplementary material available at 10.1186/s13019-022-02080-6.

## Introduction

T(A)AD (Stanford type A aortic dissection) is one of the most catastrophic emergencies of the cardiovascular system and is an important cause of sudden death [1, 2]. According to the current literature, the incidence of T(A)AD in different countries and regions ranges from 2.1 to 16.3 per 100,000 persons [3, 4]. Simultaneously, T(A)AD in acute phase has a total case-fatality rate of 73% and a pre-hospital mortality rate of 49% [5]. Although immediate surgical intervention significantly improves survival, surgical mortality of T(A)AD patients remains high [6]. The in-hospital mortality of T(A)AD was reported as high as 22% over the last decade [7]. Due to the complex etiology and high mortality, the treatment and perioperative management of T(A)AD remain a great challenge. However, there is still a lack of systematic studies on the factors affecting the prognosis of T(A)AD during hospitalization.

Obesity is recognized as a common chronic morbidity. It has a high prevalence, which is still on an upward trend, among both male and female population globally [8]. The average life expectancy of the population has also been on the rise over the last two–three decades. On the other hand, this means that the adverse metabolic effects of obesity could remain even for a longer time, thus increasing the risk of obesity-related diseases such as type 2 diabetes mellitus, stroke, coronary heart disease and hypertension [9, 10]. Also, people with a BMI of 40 or greater can be further diagnosed as morbid obese, which poses a greater challenge to the survival of patients with heart disease, stroke and many cancers [11–13]. Cardiovascular disease is particularly impacted by morbid obesity, as is T(A)AD [14]. Morbid obesity is relevant to more complications, such as acute lung injury (ALI) and hypoxemia in T(A)AD patients [15]. Also, bariatric surgery is associated with a lower likelihood of admission for aortic dissection [16]. Few studies have concentrated specifically on in-hospital outcomes of T(A)AD inpatients with morbid obesity. A recent clinical study showed that obesity was not a risk of death or other adverse outcomes for the patients undergoing surgical repair of T(A)AD [17], while the effects of morbid obesity are still unknown. Such single-center studies could not be able to provide enough evidence for the impact of morbid obesity due to small sample size.

The aim of this study is to determine whether morbid obesity contributes to higher in-hospital mortality and healthcare resource utilization in patients with T(A)AD in the United States by using the Nationwide Inpatient Sample (NIS). A better understanding of the relationship between morbid obesity and T(A)AD may be an essential step in reducing mortality as well as economic burden in hospitalized patients with T(A)AD.

## Methods

### Data source

Data from this study was obtained from the NIS, which is a portion of the Healthcare Cost and Utilization Project and one of the largest all-payer inpatient health care databases. This database was a publically available database and is available to everyone. The information from NIS can be used to make national estimates of health care utilization, charges, quality, as healthcare resource use and clinical characteristics are included. Begin from 2012, the NIS went through a redesign aimed at representing over 95% of the US population. Detailed information is available according to the official website https://www.hcup-us.ahrq.gov, and on account of no patient-identifiable information, approval from ethical institutions is not required.

### Study population

**Fig. 1:**
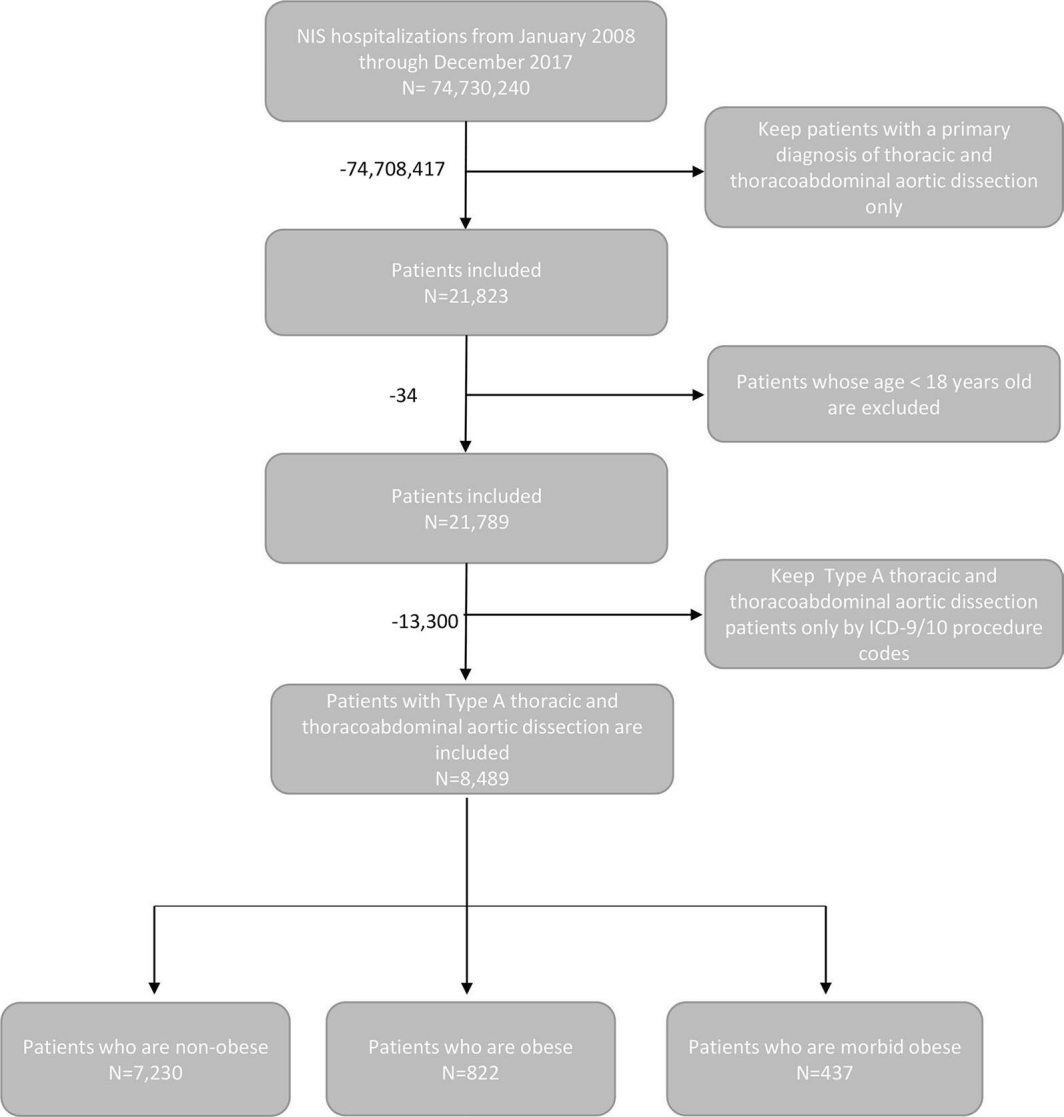
The flowchart of the cohort selection process

All patients aged ≥ 18 years with a primary diagnosis of T(A)AD from 2008 to 2017 were included. First of all, we assessed primary diagnosis variable to identify TAD (Thoracic Aortic Dissection) inpatients by utilizing the Clinical Modification diagnosis code (ICD-9-CM) 441.01, 441.03 and ICD-10-CM diagnosis code I71.01, I71.03. Then we assessed every procedure variable to distinguish T(A)ADs using criteria developed by Sachs et al. [18], with procedure codes for cardioplegia, valve repair, or operations on vessels of the heart, cardiopulmonary bypass or hypothermia which were exclusively performed on T(A)AD inpatients to a great extent. List of ICD-9/10 procedure codes for locating T(A)AD patients are listed in Additional file 1: Table S1. Considering that the WHO classifies obesity into class I (body mass index (BMI) of 30–34.99 kg/m^2^), class II (BMI of 35–39.99 kg/m^2^) and class III (morbid obesity; BMI of ≥ 40 kg/m^2^), we further grouped patients into non-obese, obese (BMI of 30–39.99 kg/m^2^) and morbid obese (BMI of ≥ 40 kg/m^2^). Then we identified BMI categories or obesity status of patients by non-obesity, obesity (V85.30-39, 278.00) and morbid obesity (V85.40-45, 278.01) based on ICD-9-CM codes and by obesity (Z68.30-39, E66.8, E66.9, E66.09), morbid obesity (Z68.40-45, E66.01) based on ICD-10-CM codes, according to previous document researches [19]. The process of the cohort selection was shown in Fig. 1 (p. 28).

### Covariates and outcome measures

Patient-related variables from NIS were recorded including sex, age, race, elective admission or not, insurance type, income status, smoking, dyslipidaemia, coronary artery disease, prior stroke, long-term anticoagulants and antithrombotics and Elixhauser Comorbidity Index (ECI) which contains common comorbidities and was calculated to assess the severity of comorbidities for each admission. Hospital characteristics like location/teaching status, bed size, and region are also included. All the covariates are given in the first column of Additional file 2: Table S2.

The primary outcomes were the prevalence of morbid obesity in T(A)AD hospitalizations and the association between morbid obesity and in-hospital mortality, total cost. Total cost was derived from total charges in the NIS database by the use of cost-to-charge ratio and Consumer Price Index (CPI) [19]. The CPI was collected from the information published by the U.S. Department of Labor. Secondary outcomes included the association between related clinical factors and obesity/morbid obesity in T(A)AD inpatients involving patient-related and hospital-level variables.

### Statistical analysis

Weighting and stratification methods are applied to obtain total national estimates, as the NIS database is based on a complex sampling design. To summarize the baseline characteristics of patients, we performed the analysis of variance and Kruskal–Wallis tests to compare distributions of continuous variables and used the chi-square test to compare differences in categorical variables.

The Cochran-Armitage trend test was conducted to analysis the temporal trend in prevalence of obesity and morbid obesity in T(A)AD patients and Cochran–Mantel–Haenszel test was used to analysis the trend of in-hospital mortality among three different weight categories. In order to evaluate the association between different obesity classes and in-hospital mortality, multivariable-adjusted logistic analyses are conducted by controlling potential confounders. Because of the right skewed distribution, we performed logarithmic transformation for total cost before using multivariable linear models. Furthermore, patient-level subgroup analyses were also explored through multivariable-adjusted logistic analyses.

Besides, we utilized univariate logistic analysis (model 1) and a multivariable-adjusted logistic analysis (model 2) to assess clinical factors associated with obesity and morbid obesity in T(A)ADs. The variables included in model 2 are clinically relevant variables based on the literature search and significant variables on univariate regression model 1 including age, sex, race, dyslipidaemia, long-term anticoagulants and antithrombotics, smoking, alcohol abuse, deficiency anemia, chronic blood loss anemia, congestive heart failure, depression, diabetes, drug abuse, hypertension, hypothyroidism, lymphoma, fluid and electrolyte disorders, renal failure. We also utilized the other two multivariable-adjusted logistic analyses (Model 3 and Model 4) as the sensitivity analyses. Age, sex, race, year of discharge, income level, insurance type, elective admission or not, hospital status and clinical factors involving smoking, prior stroke, dyslipidaemia, coronary artery disease, long-term anticoagulants and antithrombotics and 28 individual ECI comorbidities are adjusted for model 3. There's only one difference between the two models (Model 3 and Model 4) is that Model 4 uses the comorbidities score (ECI score) as a variable to estimate the outcome, which is one of the most recommended models at present (HCUP officially provides response variables).

We filled the missing data by the dominant category for categorical variables and the median for continuous variables referring to the previous studies [20] and the missing rate of all variables baseline characteristics have been reported in the Additional file 3: Table S3. R software (version 4.0.3) and the SAS version 9.4 (SAS Institute Incorporation, Cary, North Carolina, USA) are used for statistical analyses. Statistical significance was defined as a *P* < 0.05 on two-tailed testing.

## Results

### Baseline characteristics

From the NIS database 42267 T(A)AD patients were identified from January 2008 to December 2017. 36,000 (85.2%) patients were non-obese, 4095 (9.7%) were obese and 2172 (5.1%) were morbid obese (Table 1; p. 22) after weighted. Morbid obesity patients tended to be female, younger and had lower income, and they occupied the highest proportion when ECI ≥ 4. Detailed information of comorbidities showed patients with morbid obesity had the highest proportion of congestive heart failure, diabetes with or without chronic complications, fluid and electrolyte disorders and renal failure. Nevertheless, the morbid obesity had lowest proportion of rheumatoid arthritis/collagen vascular diseases. Moreover, for obese inpatients, they had the highest proportion of hypertension, dyslipidemia, and long-term anticoagulants and antithrombotics (Table 1).

**Table 1:** Baseline characteristics of T(A)AD patients by weight category

	Overall	Non-obesity	Obesity	Morbid obesity	*p*
n (%)	422667	36,000 (85.2)	4095 (9.7)	2172 (5.1)	
Age (%)					< 0.001
18–44	6293 (14.9)	5017 (13.9)	650 (15.9)	626 (28.8)	
45–64	19,577 (46.3)	16,259 (45.2)	2215 (54.1)	1103 (50.8)	
65–74	9547 (22.6)	8334 (23.2)	864 (21.1)	349 (16.1)	
≥ 75	6850 (16.2)	6390 (17.8)	366 (8.9)	94 (4.3)	
Female (%)	13,665 (32.3)	11,504 (32.0)	1317 (32.2)	844 (38.9)	0.011
Insurance type (%)					< 0.001
Medicare	16,646 (39.4)	14,735 (40.9)	1269 (31.0)	642 (29.5)	
Medicaid	4924 (11.7)	4214 (11.7)	426 (10.4)	285 (13.1)	
Private insurance	15,853 (37.5)	13,047 (36.2)	1838 (44.9)	969 (44.6)	
Self-pay	4844 (11.5)	4005 (11.1)	562 (13.7)	277 (12.7)	
Race (%)					< 0.001
White	24,622 (58.3)	21,108 (58.6)	2429 (59.3)	1085 (50.0)	
Black	7541 (17.8)	6152 (17.1)	801 (19.6)	589 (27.1)	
Hispanic	2384 (5.6)	1965 (5.5)	265 (6.5)	155 (7.1)	
Other	7720 (18.3)	6776 (18.8)	601 (14.7)	344 (15.8)	
Year (%)					< 0.001
2008	3588 (8.5)	3254 (9.0)	264 (6.4)	70 (3.2)	
2009	4156 (9.8)	3686 (10.2)	307 (7.5)	163 (7.5)	
2010	3770 (8.9)	3402 (9.4)	208 (5.1)	160 (7.4)	
2011	3968 (9.4)	3343 (9.3)	455 (11.1)	169 (7.8)	
2012	4140 (9.8)	3415 (9.5)	500 (12.2)	225 (10.4)	
2013	4240 (10.0)	3650 (10.1)	365 (8.9)	225 (10.4)	
2014	5000 (11.8)	4105 (11.4)	580 (14.2)	315 (14.5)	
2015	5080 (12.0)	4420 (12.3)	430 (10.5)	230 (10.6)	
2016	4795 (11.3)	3855 (10.7)	585 (14.3)	355 (16.3)	
2017	3530 (8.4)	2870 (8.0)	400 (9.8)	260 (12.0)	
Income quartile (%)					0.007
0–25th	11,589 (27.4)	9702 (26.9)	1195 (29.2)	693 (31.9)	
26–50th	10,063 (23.8)	8491 (23.6)	976 (23.8)	596 (27.5)	
51–75th	10,455 (24.7)	8932 (24.8)	1025 (25.0)	498 (22.9)	
76–100th	10,159 (24.0)	8876 (24.7)	899 (22.0)	385 (17.7)	
Hospital bed size (%)					0.043
Small	1995 (4.7)	1674 (4.6)	228 (5.6)	93 (4.3)	
Medium	6536 (15.5)	5497 (15.3)	754 (18.4)	285 (13.1)	
Large	33,736 (79.8)	28,829 (80.1)	3113 (76.0)	1794 (82.6)	
Hospital region (%)					< 0.001
Northeast	8478 (20.1)	7559 (21.0)	598 (14.6)	320 (14.7)	
Midwest	10,673 (25.3)	9040 (25.1)	1058 (25.8)	576 (26.5)	
South	13,616 (32.2)	11,377 (31.6)	1427 (34.8)	812 (37.4)	
West	9501 (22.5)	8025 (22.3)	1012 (24.7)	464 (21.3)	
Rheumatoid arthritis/collagen vascular diseases	923 (2.2)	848 (2.4)	55 (1.4)	20 (0.9)	0.030
Congestive heart failure	987 (2.3)	793 (2.2)	105 (2.6)	89 (4.1)	0.034
Depression	2577 (6.1)	2075 (5.8)	367 (9.0)	136 (6.2)	0.001
Diabetes, uncomplicated	4198 (9.9)	3080 (8.6)	686 (16.8)	432 (19.9)	< 0.001
Diabetes with chronic complications	967 (2.3)	705 (2.0)	167 (4.1)	95 (4.4)	< 0.001
Drug abuse	2013 (4.8)	1793 (5.0)	174 (4.3)	45 (2.1)	0.018
Hypertension (combine uncomplicated and complicated)	31495 (74.5)	26,368 (73.2)	3422 (83.6)	1705 (78.5)	< 0.001
Fluid and electrolyte disorders	207,86 (49.2)	17,481 (48.6)	2104 (51.4)	1202 (55.3)	0.010
Renal failure	6049 (14.3)	4995 (13.9)	648 (15.8)	406 (18.7)	0.008
ECI (%)					< 0.001
0–1	7851 (18.6)	6976 (19.4)	587 (14.3)	288 (13.3)	
2	9131 (21.6)	7951 (22.1)	778 (19.0)	402 (18.5)	
3	9642 (22.8)	8117 (22.5)	997 (24.4)	528 (24.3)	
≥ 4	15,643 (37.0)	12,957 (36.0)	1733 (42.3)	954 (43.9)	
Dyslipidemia	12,750 (30.2)	10,380 (28.8)	1607 (39.3)	762 (35.1)	< 0.001
Long-term anticoagulants and antithrombotics	2661 (6.3)	2179 (6.1)	351 (8.6)	131 (6.0)	0.018
In-hospital mortality (%)	6277 (14.9)	5399 (15.0)	543 (13.3)	335 (15.4)	0.605
Total cost (median [IQR])	68,867.78 [47,392.87, 105,510.96]	68,670.58 [47,282.43, 105,163.85]	68,670.58 [47,282.43, 105,163.85]	75,647.46 [51,150.84, 120,032.79]	0.003
Length of stay (median [IQR])	10.00 [6.00, 16.00]	10.00 [6.00, 16.00]	10.00 [6.00, 16.00]	11.00 [7.00, 18.00]	0.002

T (A)AD, type A thoracic aortic dissection; IQR, interquartile range

### Association between obesity/morbid obesity and in-hospital mortality, total cost

In-hospital mortality was 13.3% and 15.4% in patients with obesity and morbid obesity respectively (Table 1), and multivariable regression analysis indicated that morbid obesity was associated with increased risk of in-hospital mortality ((odds ratio [OR], 1.39; 95% confidence interval [CI] 1.03–1.86). Median total cost was $67,541.51 (interquartile range [IQR]: $47,099.56–$102,565.05) and $75,647.46 (IQR $51,150.84–$120,032.79) in patients with obesity and morbid obesity respectively. Patients with morbid obesity was also associated with 8% higher total cost (estimate: 0.08; 95% CI 0.04–0.12; *P* < 0.001) compared with the non-obese ones (Table 2; p. 30). However, compared with non-obese patients, there was no significant association between obesity and in-hospital mortality. There was also no significant association between obesity and total cost compared with non-obese ones. (Table 2; p. 25).

**Table 2:** The association between different obesity classes and in-hospital mortality, total cost estimated by adjusted model

Primary outcomes	Non-obese	Obesity		Morbid obesity	
In-hospital mortality, n (%)	5398.9 (15.0)	543.4 (13.3)		334.7 (15.4)	
	Ref	OR (95%CI)	*P* value	OR (95%CI)	*P* value
	Ref	1.02 (0.80,1.30)	0.880	1.39 (1.03,1.86)	0.030
Total cost, median [IQR]	68670.58 [47282.43, 105163.85]	67541.51 [47099.56, 102565.05]		75647.46 [51150.84, 120032.79]	
	Ref	Estimate (95%CI)	*P *value	Estimate (95%CI)	*P *value
	Ref	− 0.02 (− 0.06,0.01)	0.146	0.08 (0.04,0.12)	< 0.001

Variables selected in the model for adjustment included age, sex, race, year of discharge, income level, insurance type, elective admission or not, hospital factors and clinical factors involving smoking, prior stroke, dyslipidaemia, coronary artery disease, long-term anticoagulants and antithrombotics and 28 individual ECI comorbidities

OR, odds ratio; CI, confidence interval

### Trends of obesity/morbid obesity prevalence and in-hospital mortality

**Fig. 2:**
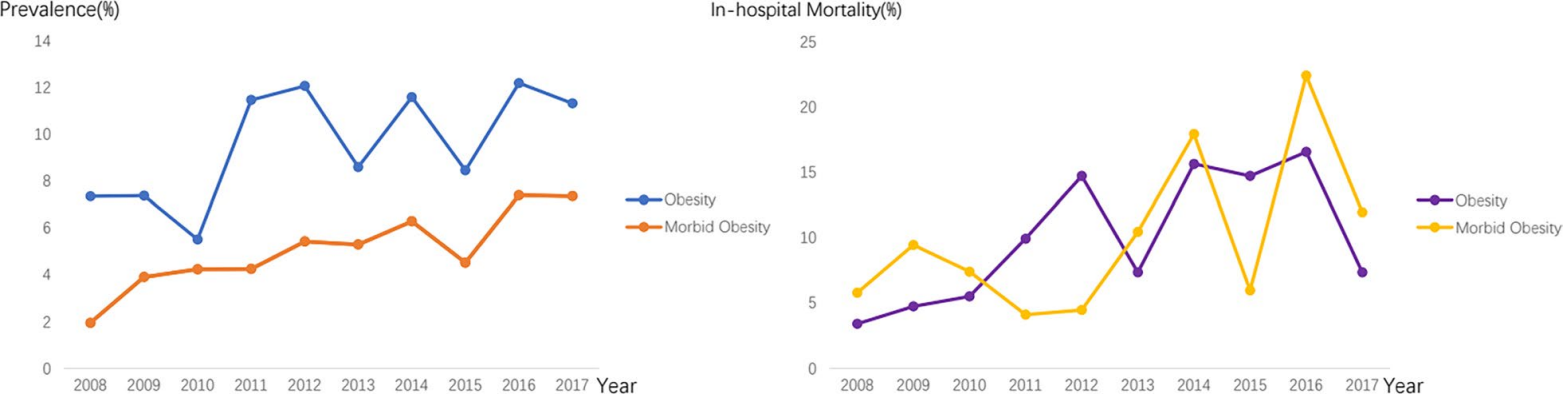
Temporal trends of obesity and morbid obesity prevalence in T(A)AD inpatients and in-hospital mortality in T(A)AD patients with different weight categories. T(A)AD, type A thoracic aortic dissection

From 2008 to 2017, the rate of obesity and morbid obesity in patients with T(A)AD have significantly increased from 7.36 to 11.33% and from 1.95 to 7.37% (*P for trend test* < 0.001; Fig. 2; p. 29). Furthermore, the rate of in-hospital mortality in inpatients with obesity and morbid obesity both have grossly increased from 3.42 to 7.36% and from 5.80 to 11.95% (*P for trend test* < 0.001; Fig. 2). The highest mortality rate occurred in 2016 was 22.4% (Fig. 2).

### Association between clinical factors and obesity/morbid obesity among T(A)AD inpatients

Factors associated with morbid obesity included age (OR 0.07; 95% CI 0.04–0.11), female (OR 1.66; 95% CI 1.34–2.06), dyslipidemia (OR 1.59; 95% CI 1.26–1.99), congestive heart failure (OR 1.80; 95% CI 1.04–3.14), diabetes (OR 2.83; 95% CI 2.15–3.73), drug abuse (OR 0.30; 95% CI 0.15–0.60) and fluid and electrolyte disorders (OR 1.27; 95% CI 1.03–1.56) among T(A)AD hospitalizations. Moreover, age (OR 0.29; 95% CI 0.21–0.40), dyslipidemia (OR 1.50; 95% CI 1.26–1.77), depression (OR 1.40; 95% CI 1.08–1.82), diabetes (OR 2.05; 95% CI 1.66–2.54) and hypertension (OR 1.68; 95% CI 1.36–2.06) were also connected with higher risk of obesity in T(A)ADs (Table 3; p. 26 and Additional file 5: Figure S1).

**Table 3:** Association between clinical factors and obesity/morbid obesity among T(A)AD inpatients

Variables	Effect	Non-obese	Univariate model 1				Multivariable model 2^a^			
			Obesity		Morbid obesity		Obesity		Morbid obesity	
		Ref	OR (95% CI)	*P *value	OR (95% CI)	*P *value	OR (95% CI)	*P *value	OR (95% CI)	*P *value
Age	18–44 versus 45–64	Ref	1.05 (0.85,1.30)	0.636	0.54 (0.44,0.68)	< 0.001	0.87 (0.70,1.08)	0.212	0.44 (0.35,0.56)	< 0.001
	18–44 versus 65–74	Ref	0.80 (0.62,1.03)	0.086	0.34 (0.25,0.45)	< 0.001	0.55 (0.42,0.72)	< 0.001	0.21 (0.15,0.29)	< 0.001
	18–44 versus ≥ 75	Ref	0.44 (0.32,0.60)	< 0.001	0.12 (0.07,0.19)	< 0.001	0.29 (0.21,0.40)	< 0.001	0.07 (0.04,0.11)	< 0.001
Sex	Male–female	Ref	1.01 (0.87,1.17)	0.898	1.35 (1.11,1.65)	0.003	1.13 (0.97,1.32)	0.118	1.66 (1.34,2.06)	< 0.001
Race	White–Black	Ref	1.13 (0.93,1.37)	0.209	1.86 (1.46,2.37)	< 0.001	0.91 (0.75,1.11)	0.366	1.28 (0.98,1.68)	0.075
	White–Hispanic	Ref	1.17 (0.85,1.60)	0.327	1.53 (1.04,2.25)	0.031	0.94 (0.69,1.29)	0.702	1.01 (0.66,1.54)	0.970
	White–other	Ref	0.77 (0.61,0.98)	0.034	0.99 (0.74,1.32)	0.924	0.73 (0.58,0.93)	0.011	0.84 (0.62,1.15)	0.274
Dyslipidemia		Ref	1.59 (1.37,1.86)	< 0.001	1.33 (1.10,1.62)	0.004	1.50 (1.26,1.77)	< 0.001	1.59 (1.26,1.99)	< 0.001
Congestive heart failure		Ref	1.17 (0.72,1.88)	0.527	1.90 (1.16,3.12)	0.011	1.26 (0.78,2.05)	0.349	1.80 (1.04,3.14)	0.037
Depression		Ref	1.61 (1.25,2.07)	< 0.001	1.09 (0.72,1.64)	0.686	1.40 (1.08,1.82)	0.011	1.02 (0.67,1.56)	0.923
Diabetes, uncomplicated		Ref	2.15 (1.76,2.63)	< 0.001	2.65 (2.07,3.41)	< 0.001	2.05 (1.66,2.54)	< 0.001	2.83 (2.15,3.73)	< 0.001
Diabetes with chronic complications		Ref	2.13 (1.40,3.23)	< 0.001	2.29 (1.42,3.71)	0.001	2.23 (1.45,3.42)	< 0.001	2.45 (1.46,4.10)	< 0.001
Drug abuse		Ref	0.85 (0.59,1.22)	0.376	0.41 (0.21,0.79)	0.008	0.75 (0.51,1.11)	0.149	0.30 (0.15,0.60)	< 0.001
Hypertension (combine uncomplicated and complicated)		Ref	1.86 (1.52,2.26)	< 0.001	1.34 (1.06,1.68)	0.014	1.68 (1.36,2.06)	< 0.001	1.24 (0.97,1.59)	0.089
Fluid and electrolyte disorders		Ref	1.12 (0.96,1.31)	0.151	1.31 (1.07,1.60)	0.008	1.12 (0.96,1.31)	0.155	1.27 (1.03,1.56)	0.027

OR, odds ratio; CI, confidence interval; T(A)AD, type A thoracic aortic dissection

^a^Multivariable model 2: adjusted for age, sex, race, dyslipidaemia, long-term anticoagulants and antithrombotics, smoking, alcohol abuse, deficiency anemia, chronic blood loss anemia, congestive heart failure, depression, diabetes, drug abuse, hypertension, hypothyroidism, lymphoma, fluid and electrolyte disorders, renal failure.

### Sensitivity analyses

Sensitivity analyses also showed that clinical factors associated with morbid obesity included age, female, dyslipidemia, diabetes, drug abuse. Age, dyslipidemia, depression, diabetes and hypertension were also associated with higher risk of obesity in T(A)ADs (Additional file 4: Table S4).

### Subgroup analyses

As shown in Table 4 (p. 27), morbid obesity had association with in-hospital mortality in male (OR 1.65; 95% CI 1.15–2.37) rather than female as well as patients who had fluid and electrolyte disorders (OR 1.61; 95% CI 1.09–2.36). Besides, morbid obesity in patients who did not have smoking history (OR 1.61; 95% CI 1.16–2.23) or did not have comorbidities as drug abuse (OR 1.41; 95% CI 1.04–1.90), dyslipidemia (OR 1.65; 95% CI 1.15–2.35) or diabetes (OR 1.41; 95% CI 1.05–1.91) were associated with higher risk of in-hospital mortality. For the youngest age group, T(A)AD inpatients with morbid obesity were connected with higher risk of hospital mortality (OR 2.81; 95% CI 1.50–5.24).

**Table 4:** The association between different weight categories and in-hospital mortality in subgroups of T(A)AD inpatients

Subgroup	In-hospital Mortality (Non-obese)	In-hospital Mortality (Morbid obesity)		Non-obese n (%)	Morbid obesity n (%)
	Ref	OR (95%CI)	*P *value		
Sex					
Male	Ref	1.65 (1.15,2.37)	0.007	3427 (14.0)	214 (16.1)
Female	Ref	0.99 (0.59,1.67)	0.970	1972 (17.2)	121 (14.3)
Age					
18-44	Ref	2.81 (1.50,5.24)	0.001	433 (8.6)	114 (18.3)
45-64	Ref	1.00 (0.63,1.57)	0.991	2026 (12.5)	145 (13.2)
65-74	Ref	1.10 (0.49,2.46)	0.824	13,569 (16.3)	45 (13.0)
≥75	Ref	2.10 (0.64,6.87)	0.220	1583 (24.8)	30 (31.8)
Smoke					
No	Ref	1.61 (1.16,2.23)	0.004	4094. (16.3)	275 (17.3)
Yes	Ref	0.77 (0.37,1.63)	0.497	1305 (12.0)	60 (10.3)
Drug abuse					
No	Ref	1.41 (1.04,1.90)	0.025	5209 (15.2)	330 (15.5)
Yes	Ref	0.34 (0.03,4.18)	0.401	190 (10.6)	5 (11.1)
Dyslipidemia					
No	Ref	1.65 (1.15,2.35)	0.006	3991 (15.6)	259 (18.4)
Yes	Ref	0.95 (0.53,1.71)	0.855	1408 (13.6)	76 (9.9)
Diabetes					
No	Ref	1.41 (1.05,1.91)	0.023	4920 (15.0)	276 (15.9)
Yes	Ref	0.90 (0.44,1.81)	0.760	479 (15.6)	59 (13.6)
Fluid and electrolyte disorders					
No	Ref	1.22 (0.76,1.96)	0.419	2522 (13.6)	116 (12.0)
Yes	Ref	1.61 (1.09,2.36)	0.016	2877 (16.5)	219 (18.2)

T(A)AD, type A thoracic aortic dissection

## Discussion

This contemporary analysis is the largest nationwide study focusing on effects of obesity and morbid obesity on T(A)AD inpatients. In our studies, morbid obesity was related to higher risk of in-hospital mortality among T(A)ADs and T(A)AD inpatients with morbid obesity burdened higher total cost compared to non-obese group. We also found an increasing prevalence of both obesity and morbid obesity in T(A)AD patients from 2008 to 2017. Significant proportion of morbid obesity was observed in the youngest age group, female and elective admission population. Meanwhile, age, sex, dyslipidemia, congestive heart failure, diabetes, drug abuse and fluid and electrolyte disorders were also relevant to morbid obesity in T(A)AD patients.

Obesity has many adverse effects on the general population, particularly on cardiovascular health. Although obesity has been identified as an independent risk factor for many cardiovascular diseases, evidence from several previous clinical cohorts suggested an obesity paradox (OP) that obese patients with cardiovascular diseases tend to have an improved short and long-term prognosis [21]. Analysis of ACS-NSQIP data on cardiovascular surgeries indicated that patients with body mass index (BMI) 25–40 kg/m^2^ experienced lower odds of 30-day mortality following abdominal aortic aneurysm repair [22]. A multivariate analysis of 78,762 Canadian patients with coronary artery bypass grafting (CABG) and CABG combined with aortic valve replacement found a survival advantage for patients with BMI 25–29.9 kg/m^2^ compared with normal weight patients. Limited increases in BMI may provide a survival benefit, however the protective effect seems to decline when severe or morbid obesity is present. Currently, there are still few large-sample studies on the impact of obesity or morbid obesity on the perioperative prognosis of patients with T(A)AD.

In our study, morbid obesity was a significant risk factor for in-hospital mortality of T(A)AD patients (OR 1.39; 95% CI 1.03–1.86), while obesity was not. This finding was inconsistent with the hypothesis of OP and led to more attention being paid to T(A)AD patients with morbid obesity. Comorbidities of morbid obesity might explain the higher mortality, such as congestive heart failure, diabetes, fluid and electrolyte disorders and renal failure, which were demonstrated in the baseline characteristics. Previous studies had shown that morbid obesity contributed to an increased incidence of preoperative hypoxemia in patients with T(A)AD, and a higher incidence of post operation ALI was also observed, which remains a major complication and the leading cause of death after thoracic surgery [15]. It was also reported that time to extubating of patients with T(A)AD was significantly delayed by morbid obesity [17]. Although various factors have been postulated for the better outcome in obese patients including lower incidence of undernutrition, possible presence of protective cytokine, and greater metabolic reserves, the poorer outcomes in morbidly obese patients suggest that with progression of obesity, there remains an increasing risk of death.

Subgroup analyses also indicated some high-risk population such as male patients. Men themselves are at high risk of T(A)AD [14], thus weight management could be particularly important for them. Interestingly, despite a higher prevalence of morbid obesity in female patients with T(A)AD, morbid obesity was not a risk factor for in-hospital mortality in them. Morbid obesity significantly increased in-hospital mortality in the youngest age group (18–44 years old), but not in the groups over 45 years of age. It was reported that proximal and distal progression of dissection occurred more frequently in younger T(A)AD patients. Meanwhile, age was negatively correlated with the incidence of Valsalva sinus level intimal tear, which led to the choice of more radical proximal surgical repair in younger patients [23]. Morbid obesity may be associated with the worse prognostic implications of these surgeries. The non-diabetic was shown to be more dangerous than the diabetic, which is consistent with the latest finding that diabetes may be a protective factor for aortic dissection [24]. Besides, patients without dyslipidemia, smoking or drug abuse were at higher risk, which was unexpected. Earlier and better disease management, such as the use of statins and antihypertensive drugs, may help improve outcomes in T(A)AD patients with dyslipidemia. This warrants further investigation.

The prevalence of obesity exceeds 30% in the USA, posing a critical public health concern as well as a huge financial burden. In 2014, the economic impact of obesity was estimated to be 2.8% of the global gross domestic product (GDP) [25]. The medical costs of obesity have been found to exceed those of smoking in the USA, and severe obesity doubles these excess costs [26]. Not unlike the extensive acceptance of the dangers of smoking, obesity, while increasing, also requires public attention. As is shown in our study, morbid obesity was associated with higher total cost. Morbid obesity made even a heavier financial burden for T(A)AD patients since these patients tended to have lower income. Cost of illness (COI) studies help policy makers better understand the economic burden of specific diseases. At the macro level, our findings contribute to the development and prioritization of health care policy and the allocation of health care resources. On a personal level, it also helps to emphasize the necessity of weight loss for morbidly obese patients.

In terms of trends, there was a significant increase in the prevalence of obesity from 7.36% to 11.33% among T(A)AD inpatients from 2008 to 2017. Morbid obesity prevalence trends in T(A)AD patients may be of more concern (from 1.95 to 7.37%). Increasing obesity burden in the US suggests a growing challenge for T(A)AD management. Several centers have reported an overall incident of 4.6–7.6 per 100,000 for T(A)AD consistently with an increasing trend over time [27]. Due to improvements in detection, appropriate rapid transfers, and attentive management of patients, the annual number of operations rates also continues to increase [28]. An encourage finding is that, according to most centers, the operation mortality of T(A)AD is in a falling trend, which is attributed to improving surgical techniques and better perioperative management. As is demonstrated in our research, long-term or even lifelong weight management for T(A)AD patients could be wise, considering the rapid increase in the prevalence of morbid obesity. Given that verbal advice often has little effect, bariatric surgery may be worth considering for some high-risk patients.

## Limitations

This study is subject to limitations. Firstly, although previously validated codes were used for T(A)AD and morbid obesity, potential under coding and coding errors are inevitable. Particularly since we identified the patients with common procedures which are carried out in T(A)AD as there is no inherent code which can identify the T(A)AD patients directly, it may generate biased results and lead to inappropriate identification of patients, even though it was published previously by Sachs et al. ^18^. However, in the context of large samples, these errors are more likely to be random. Secondly, the NIS database could not identify readmissions for T(A)AD patients. Readmission rates of T(A)AD patients with and without obesity were unclear. Considering that most postoperative complications of T(A)AD occur in-hospital and only a small proportion of patients require readmission for further treatment, which is acceptable given the adequate sample size. Besides, NIS database is a de-identified database that cannot trace individual records and lacks post-discharge or long-term outcomes. Further prospective studies are required to elucidate the risk profile over time and long-term outcomes in T(A)AD patients with morbid obesity. Last but not the least, this study is a retrospective observational analysis, where cofounding bias might exist and have affected our results. Nevertheless, univariate and multivariable logistic regression were both applied to balance covariates and reduce the influence of confounding factors.

## Conclusions

In conclusion, morbid obesity is associated with worse clinical outcomes and more health resource utilization in T(A)AD patients. The prevalence of obesity and morbid obesity in hospitalized T(A)AD patients has continued to increase from 2008 through 2017. In order to reverse this situation, appropriate medical resource orientation and weight management education for T(A)AD patients may be necessary.

## Supplementary Information

**Additional file 1. Table S1: **List of ICD-9/10 procedure codes for identifying T(A)AD patients and other other common comorbidities.

**Additional file 2. Table S2: **Baseline characteristics of T(A)AD patients by weight category.

**Additional file 3. Table S3: **Missing values of baseline characteristics in T(A)Ads.

**Additional file 4. Table S4: **Association between clinical factors and obesity/morbid obesity among T(A)AD inpatients.

**Additional file 5. Figure S1: **Association between clinical factors and obesity / morbid obesity among T(A)AD inpatients.

## Data Availability

The dataset analyzed during this study are not publicly available because it was paid for use, and it cannot be disclosed without official permission.

## Abbreviations

T(A)AD: Type A aortic dissection
NIS: National Inpatient Sample Database
ALI: Acute lung injury
ECI: Elixhauser Comorbidity Index
OR: Odds ratio
CI: Confidence interval
IQR: Interquartile range
OP: Obesity paradox
BMI: Body mass index
CABG: Coronary artery bypass grafting
GDP: Gross domestic product
COI: Cost of illness
