# Supplementary figures and images for: Morbid obesity impacts mortality among inpatients with type a aortic dissection: an analysis of the national inpatient sample
Source: J Cardiothorac Surg. 2023 Jan 10;18:14. doi: 10.1186/s13019-022-02080-6 (PMC9832697; doi:10.1186/s13019-022-02080-6)

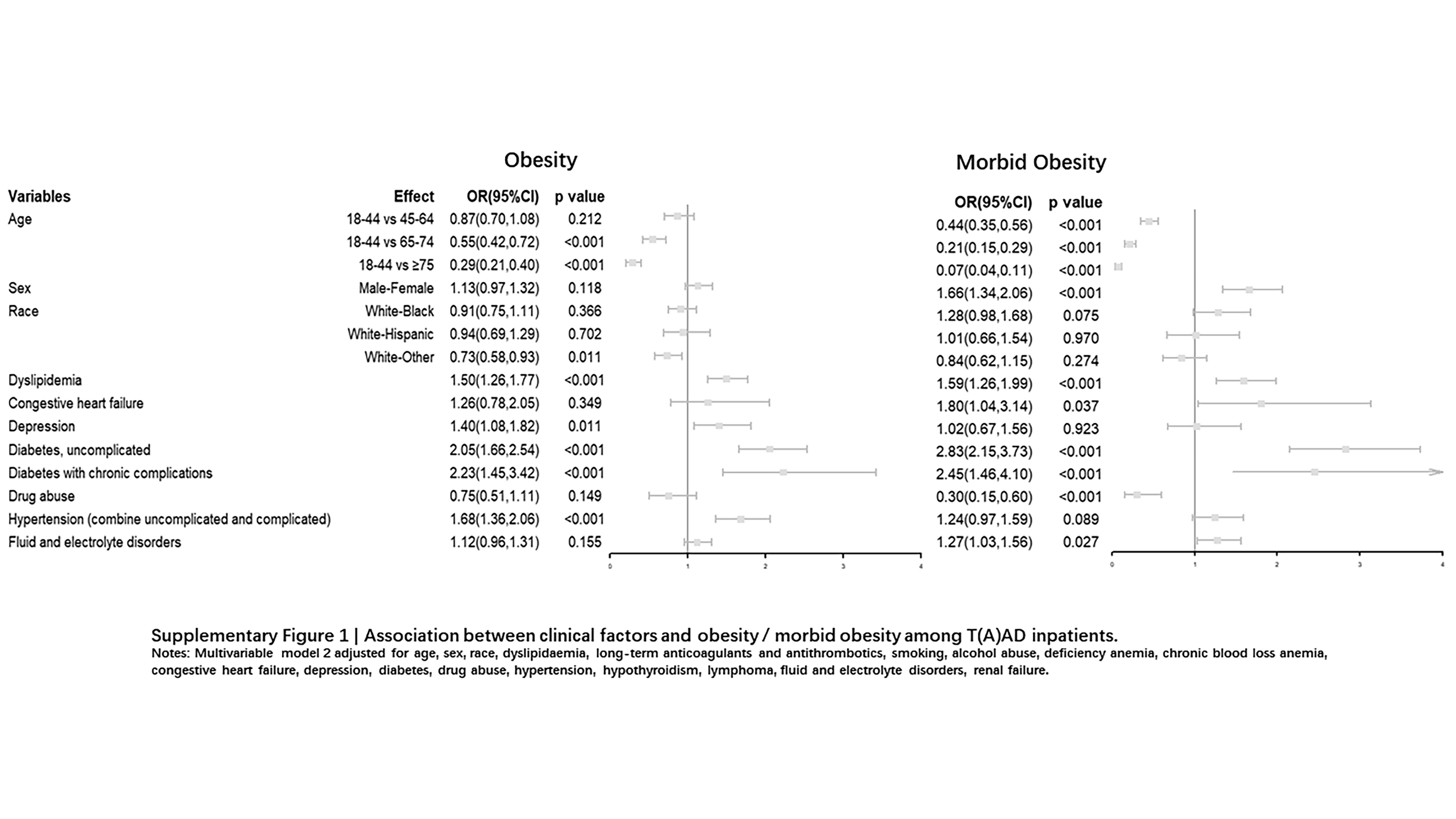

Supplement: Supplementary file 5 — Additional file 5. Figure S1: Association between clinical factors and obesity / morbid obesity among T(A)AD inpatients. [file 13019_2022_2080_MOESM5_ESM.tif]
